# Intrusive rumination and academic burnout among adolescents in ethnic minority areas of China during the COVID-19 pandemic: PTSS as mediator and cognitive reappraisal as moderator

**DOI:** 10.1186/s12889-023-17133-1

**Published:** 2023-11-08

**Authors:** Linhui He, Xiaojiao Yuan, Qiuyan Chen, Xiaogang Wang

**Affiliations:** 1 School of Education and Psychology, Southwest Minzu University, Chengdu, China; 2 Key Research Institute of Humanities and Social Sciences of State Ethnic Affairs Commissionin, Southwest Minzu University, Chengdu, 610225 China

**Keywords:** Adolescents in ethnic minority areas, Intrusive rumination, Academic burnout, PTSS, Cognitive reappraisal, COVID-19

## Abstract

**Background:**

The COVID-19 pandemic has had a significant negative impact on public health, prompting scholarly research in related fields. In this context, the present study reveals the psychological characteristics of adolescents in ethnic minority areas of China approximately five months after the 2020 outbreak of the COVID-19 pandemic, explores the relationship between intrusive rumination and academic burnout, and examines the role of post-traumatic stress symptoms (PTSS) and cognitive reappraisal in the relationship to provide an empirical foundation for developing effective psychological interventions for adolescents in the wake of the pandemic.

**Methods:**

Based on cluster sampling, 941 middle school students (65.36% female, 74.71% senior high, M_age_=15.95) in ethnic minority areas of China were surveyed using the Event Related Rumination Scale, Adolescent Academic Burnout Scale, Post-traumatic Stress Checklist Scale, Emotion Regulation Strategy Scale, and a self-designed demographic questionnaire.

**Results:**

During the COVID-19 pandemic, 7.44% of Chinese ethnic minority adolescents in our study sample were classified as PTSD positive, and 10.95% exhibited partial PTSD. Intrusive rumination significantly predicted academic burnout, and PTSS played a key mediating role between the two, accounting for 58.51% of the total effect. After controlling for PTSS, cognitive reappraisal moderated the effects of intrusive rumination on academic burnout. Specifically, the effect of intrusive rumination on academic burnout decreased with improvement in cognitive reappraisal.

**Conclusions:**

Intrusive rumination indirectly affected academic burnout in adolescents through PTSS as a crucial mediator, and the remnant direct effect was alleviated by cognitive reappraisal. This finding emphasises the importance of adopting a comprehensive approach that encompasses cognitive, emotional, and physiological symptoms to understand and address academic burnout among adolescents during the COVID-19 pandemic.

## Background

COVID-19 is a highly infectious disease officially declared a global pandemic by the World Health Organization on 11 March 2020 [1]. Since the outbreak, it has seriously and profoundly impacted people’s lives, triggering various adverse psychological reactions [2, 3] and behavioural responses [4]. One study indicated that nearly 35% of respondents suffered from varying degrees of psychological distress due to COVID-19 [5]. As a vulnerable group during major public health events, adolescents are particularly susceptible to the effects of the external environment [6]. During the COVID-19 pandemic, adolescents have experienced numerous stressful events, including anxiety and fear of the virus, parent-child conflict due to prolonged family isolation, and negative emotions such as discomfort from switching learning modes [7, 8]. All these factors inevitably take a toll on their lifestyle and physical and mental health [9] and, in particular, have a considerable impact on their psychological state of learning [10, 11], leading to the development or exacerbation of academic burnout [12].

Academic burnout refers to students’ negative emotions and avoidance behaviours towards learning due to academic stress [13]. Previous studies have shown that academic burnout not only leads to lower student performance [14] but also largely contributes to the development of psychological problems such as severe depression and inappropriate behaviours such as truancy [15]. Recent research has demonstrated a significant increase in student burnout during the pandemic [16]. Zeng et al. [12] surveyed ethnic minority adolescents in China and found that the level of academic burnout among adolescents in ethnic minority areas during the COVID-19 pandemic was higher than among adolescents in other non-ethnic areas in similar studies. However, mechanisms underlying academic burnout among adolescents during the COVID-19 pandemic remain unclear. This study aimed to explore the mechanism of academic burnout among adolescents in ethnic minority areas of China during the pandemic from the perspective of the interaction between cognitive, emotional regulation, and physical symptoms.

### Intrusive rumination and academic burnout

Intrusive rumination is a common cognitive pattern observed in individuals who have experienced traumatic or stressful events. This concept refers to the persistent intrusion of relevant events into an individual’s thoughts, leading to a heightened focus on negative aspects and an increased negative evaluation of those experiences [17]. According to the theory of resource allocation, negative thoughts associated with psychological distress symptoms can deplete cognitive resources, thereby impairing an individual’s ability to manage important tasks [18]. Research has consistently indicated a strong correlation between rumination and heightened academic stress [19]; similarly, studies have also reported a correlation between rumination and the development of negative emotions such as anxiety and guilt [20, 21]. These can undermine individuals’ motivation and self-confidence in their academic pursuits, leading to avoidance behaviours and potentially culminating in long-term outcomes, such as burnout [22].

During the COVID-19 pandemic, adolescents have been continuously exposed to an overwhelming amount of information about the virus and its transmission, resulting in uncontrollable worries and negative thoughts. These persistent negative thoughts may affect their thinking processes and intensify academic stress and negative emotions. Consequently, this may contribute to academic burnout, characterised by negative emotional states and a tendency to avoid academic responsibilities. Therefore, we hypothesised the following:

#### Hypothesis 1

Intrusive rumination positively predicts academic burnout among adolescents in ethnic minority areas of China during the COVID-19 pandemic.

### The mediating role of post-traumatic stress symptom

PTSS refers to a series of psychological symptoms that arise in individuals following their personal experiences or exposure to threatening events, such as major natural disasters or violent attacks. These include traumatic and avoidance symptoms, negative cognitive and emotional changes related to traumatic events, and increased alertness [23]. According to the cognitive model of PTSD [24], intrusive rumination is a negative cognitive process involving repetitive recall and reflection on traumatic events. This rumination process can lead to ongoing distress and emotional disturbance related to the trauma, thereby exacerbating PTSS [25, 26]. Longitudinal studies have indicated that intrusive rumination is associated with the severity and persistence of initial post-traumatic stress symptoms. Higher levels of intrusive rumination predict the worsening of symptoms following traumatic events and the development of PTSS [27].

Furthermore, PTSS may exacerbate academic burnout among adolescents. The emergence of a series of symptoms related to PTSD, including emotional distress and avoidance behaviours, may cause adolescents to face academic challenges and emotional and psychological turmoil. The subsequent increase in overall academic pressure among adolescents may, to some extent, foster the emergence and exacerbation of academic burnout [28]. Research indicates that students who have experienced traumatic stress reactions are more likely to encounter learning difficulties and experience a negative impact on their academic performance [29, 30]. This outcome may erode their self-confidence, leading to reluctance to participate in learning-related activities [31], further fueling avoidance-based academic burnout [32]. Therefore, we hypothesised the following:

#### Hypothesis 2

PTSS mediates the relationship between intrusive rumination and academic burnout among adolescents in ethnic minority areas of China during the COVID-19 pandemic.

### The moderating role of cognitive reappraisal

In addition to cognitive processes, emotional processes may impact adolescent academic burnout. In the emotional processes, cognitive reappraisal is regarded as a positive and effective emotion regulation strategy. Cognitive reappraisal refers to the ability of individuals to alter their past perceptions of emotional events, create new understandings, and view them from a more positive perspective [33]. It can effectively mitigate emotional exhaustion and the resulting health issues. Numerous empirical studies have demonstrated that the use of cognitive reappraisal strategies can help traumatised individuals improve quality of life [34, 35], alleviate PTSS [36], and reduce academic burnout [28].

Emotion regulation strategies may moderate the relationship between intrusive rumination and academic burnout in adolescents. According to the broken world hypothesis, individuals often perceive the world as negative, meaningless, and worthless after a traumatic event, resulting in negative cognitive and emotional changes [37]. Intrusive rumination is a negative cognitive process associated with trauma. Previous research has shown that individuals who have experienced trauma must focus on their perception of the trauma [24, 38], with the key process of healing being the reconstruction of meaning and self-awareness in life [39]. Cognitive reappraisal, as a positive self-cognition construction process, helps individuals reevaluate their experiences and emotions from a positive perspective. This is important for ameliorating the negative impacts of intrusive rumination and PTSS on individuals. Therefore, we hypothesised the following:

#### Hypothesis 3

Cognitive reappraisal negatively predicts academic burnout in adolescents and moderates the relationship between intrusive rumination and academic burnout.

### Socio-cultural background of the Wenchuan region in China

As an ethnically diverse country, China has long upheld a cultural ethos of ‘respecting differences and tolerating diversity’. The Wenchuan region, situated in southwestern China and home to the convergence of Tibetan, Qiang, Han, and other ethnic groups, has distinct social and cultural backgrounds. The local population is heavily influenced by Tibetan Buddhism and Qiang folk beliefs that permeate all aspects of their lives. Tibetan Buddhism emphasises optimism and open-mindedness [40]. Buddhist intervention has enabled the Tibetan population to approach issues of life and death more peacefully and enhance their spiritual well-being [41]. Qiang folk beliefs emphasise fearlessness and reverence for nature and promote a conformist attitude towards the arrangement of destiny [42]. In the Qiang population, folk beliefs objectively play a role in psychological adjustment and in maintaining social order [43]. Previous research has demonstrated that, in emergencies, Qiang cultural values are crucial in regulating the psychological state of Qiang residents, reducing the psychological impact and denial caused by the crisis [44].

Thus, the unique cultural and social background of Wenchuan, which has been shaped over thousands of years, has instilled positive and optimistic cognitive traits and strong emotional regulation abilities in its residents. Owing to the region’s ethnic and cultural distinctiveness, adolescents in Wenchuan may possess unique psychological characteristics that differ from those of their peers in other regions during the COVID-19 pandemic.

In light of the foregoing, the present study will take adolescents in Wenchuan, China, as the research object, aiming to shed light on the features of intrusive rumination, academic burnout, PTSS, and cognitive reappraisal in Wenchuan against the backdrop of the COVID-19 pandemic, and seek to probe the mechanism by which intrusive rumination influences academic burnout among adolescents. This study aimed to enrich research on the public and mental health of adolescents in ethnic minority areas and provide empirical support and practical recommendations for alleviating the psychological and behavioural problems of adolescents in such areas in China during the pandemic.

**Fig. 1:**
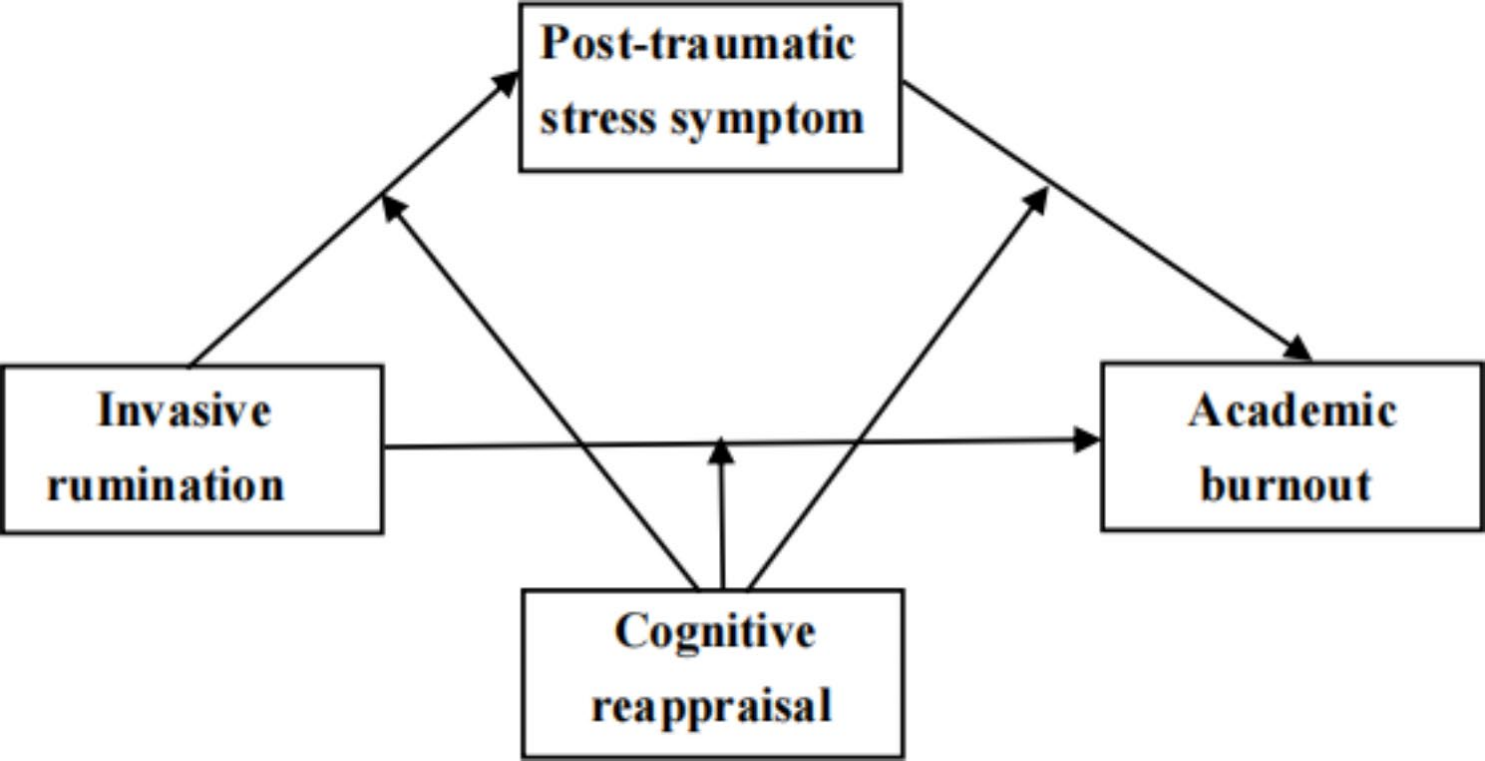
Conceptual model of the study

The conceptual framework of the study is presented in Fig. 1.

## Methods

### Participants

Cluster sampling was used to select 982 adolescents from a middle school in the Wenchuan region of China. Respondents who completed the questionnaires within an insufficient period were excluded. A total of 941 valid questionnaires were included in the study, resulting in an effective rate of 95.82%. The participants comprised 326 males (34.64%) and 615 females (65.36%), including 238 junior high school students (25.29%) and 703 senior high school students (74.71%). The sample comprised 435 Qiang (46.23%), 356 Tibetan (37.83%), 110 Han (11.69%), and 39 other minority groups (4.14%). The ethnicity information of one participant was missing. The average age of the participants was 15.95 ± 1.95years.

### Procedure

The data for this study were collected online approximately 4–5 months after the COVID-19 outbreak in China and 1.5 months before the spring semester’s final exams. Owing to the prevention and requirements of pandemic control, all schools were closed for several months, and students were at home with internet access; therefore, the study data were collected online. Mental health teachers distributed the questionnaires online after obtaining informed consent from the school directors, students, and their parents.

### Measures

#### Intrusive rumination

Intrusive rumination of adolescents during the COVID-19 pandemic was measured using the Intrusive Rumination Subscale of the Event-Related Rumination Scale [45]. It consists of ten items in total. Sample questions included, ‘Thoughts related to the COVID-19 pandemic frequently intrude into my mind and are difficult to stop’. All items were rated from 0 (*Not at all*) to 3 (*Always*). Respondents were instructed to indicate how often they felt this way in the last two weeks. Higher scores indicated higher levels of intrusive rumination. This scale has been used with ethnic groups of adolescents with good reliability and validity [46]. Cronbach’s alpha for this measure in the present study was 0.95.

#### Academic burnout

We used the Adolescent Academic Burnout Scale to measure academic burnout among adolescents during the COVID-19 pandemic, we utilised the Adolescent Academic Burnout Scale [47]. It consists of twenty-one items. Sample questions, such as ‘Studying makes me feel physically uncomfortable’, were rated on a five-point Likert scale ranging from 1 (*Never before*) to 5 (*Always*). After the dimension of low efficacy for learning was reverse-scored, higher scores on this scale indicated higher levels of academic burnout. This scale has been used with ethnic groups of adolescents with good reliability and validity [48]. Cronbach’s alpha for this measure in the present study was 0.90.

#### Post-traumatic stress symptom

We measured post-traumatic stress symptoms in adolescents during the COVID-19 pandemic using the Post-Traumatic Stress Checklist Scale [49]. It consists of seventeen items. Sample questions included, ‘I would avoid thinking about or discussing experiences related to the COVID-19 pandemic or avoid generating feelings associated with it’. These items are rated on a five-point Likert scale ranging from 1 (*Not at all*) to 5 (*Extremely*). Participants reporting higher total scores presented with higher levels of post-traumatic stress symptoms. Scores<38 on the PTSS: PTSD-negative; Scores between 38 and 49: partial PTSD; Scores ≥ 50: PTSD-positive [50]. This scale has been used in studies on ethnicity with good reliability and validity [51]. Cronbach’s alpha for this measure in the present study was 0.95.

#### Cognitive reappraisal

We used the Cognitive Reappraisal Subscale of the Emotion Regulation Scale to test adolescents’ cognitive reappraisal strategies during the COVID-19 pandemic [52]. It consists of six items. A sample question is, ‘When faced with a stressful situation, I would consider it in a way that helps me maintain composure’. These items were rated on a seven-point Likert scale from 1 (*Disagree strongly*) to 7 (*Agree strongly*). The higher the total score on the scale, the more frequently a cognitive reappraisal strategy was used. This scale has been used with ethnic minority student groups and has good reliability and validity [53]. The Cronbach’s alpha for this measure in the present study was 0.89.

#### General demographic data

Based on the needs of this study, a self-made demographic questionnaire was used to collect participants’ general information, including age, sex, grade, ethnicity, and pandemic exposure.

### Statistical approach

We used SPSS (version 25.0) for the data analysis. First, Harman’s single-factor test was used to evaluate the common method bias. The results showed seven factors with eigenvalues greater than 1. The first factor explained 27.31% of the variance, which was below the critical threshold of 40% [54], indicating no significant common method bias problem in this study. Given the substantial sample size of this study, the variables were typically considered robust. The Kolmogorov-Smirnov (K-S) test was also conducted for each variable to evaluate the normality. The findings revealed that the dependent variable, academic burnout, exhibited a normal distribution (*p* > 0.05). Moreover, the kurtosis values for the remaining variables ranged from 0.08 to 2.04, and the skewness values ranged from -0.36 to 1.44, indicating approximate adherence to a normal distribution. We then conducted descriptive analyses and bivariate correlations among all study variables. Subsequently, we used Process Macro (version 3.4) [55] to examine our main hypotheses.

## Results

### Descriptive statistics

**Table 1:** The basic profile of study variables (*M* ± *SD*)

	Intrusive rumination	Academic burnout	PTSS	Cognitive reappraisal
Female(N = 615)	0.71 ± 0.58	2.46 ± 0.57	1.68 ± 0.66	4.74 ± 0.99
Male(N = 326)	0.82 ± 0.65	2.49 ± 0.69	1.71 ± 0.71	4.59 ± 1.21
*t*	2.71^**^	0.47	0.72	-1.88
Senior high(N = 703)	0.70 ± 0.57	2.56 ± 0.58	1.69 ± 0.69	4.66 ± 1.03
Junior high(N = 238)	0.89 ± 0.69	2.21 ± 0.63	1.68 ± 0.66	4.76 ± 1.19
*t*	3.75^***^	-7.66^***^	-0.23	1.20
Han nationality(N = 110)	0.70 ± 0.57	2.36 ± 0.61	1.73 ± 0.72	4.57 ± 1.15
Tibetan(N = 356)	0.75 ± 0.61	2.46 ± 0.62	1.67 ± 0.66	4.73 ± 1.11
Qiang ethnic(N = 435)	0.73 ± 0.60	2.51 ± 0.60	1.70 ± 0.68	4.69 ± 1.01
*F*	0.35	2.60	0.31	1.06
Total(N = 941)	0.75 ± 0.61	2.47 ± 0.61	1.69 ± 0.68	4.69 ± 1.07

^**^p<0.01, ^***^p<0.001

According to the PTSD screening criteria [50], 70 adolescents (7.44%) scored ≥ 50 on the PTSS, classifying them as PTSD-positive cases; 103 adolescents (10.95%) scored between 38 and 49, categorising them as partial PTSD cases; and 768 adolescents (81.62%) scored between 17 and 38, indicating PTSD-negative cases.

Next, independent sample t-tests and one-way ANOVA were used to examine differences in intrusive rumination, academic burnout, PTSS, and cognitive reappraisal across sexes, learning stages, and ethnicities. Table 1 shows that the levels of intrusive rumination were significantly higher among male participants than among female participants (*t* = 2.71, *p* < 0.01), but no significant sex differences were found for academic burnout, PTSS, and cognitive reappraisal. Additionally, the levels of intrusive rumination were significantly higher in junior high school students than those in high school students (*t* = 3.75, *p* < 0.001), while the levels of academic burnout were significantly lower than those in high school students (*t*=-7.66, *p* < 0.001). No significant differences were found for PTSS and cognitive reappraisal across learning stages or ethnic groups.

### Correlations analysis

**Table 2:** Correlations of all variables

Variables	1	2	3	4	5	6
1. Sex	-					
2. Learning stage	0.13^***^	-				
3. Intrusive rumination	-0.09^**^	-0.13^***^	-			
4. Academic burnout	-0.02	0.25^***^	0.28^***^	-		
5. PTSS	-0.02	0.01	0.48^***^	0.44^***^	-	
6. Cognitive reappraisal	0.07^*^	-0.04	0.05	-0.23^***^	-0.09^**^	-

Sex: 0 = male; 1 = female. Learning stage: 0 = junior high; 1 = senior high

^*^p<0.05, ^**^p<0.01 and ^***^p<0.001

Table 2 displays Pearson’s correlation coefficients for all variables. Results indicate that intrusive rumination was positively associated with academic burnout and PTSS, but there was no significant correlation between intrusive rumination and cognitive reappraisal. Additionally, academic burnout was positively correlated with PTSS but negatively correlated with cognitive reappraisal. PTSS was negatively associated with cognitive reappraisal as well.

### The mechanisms of intrusive rumination on academic burnout among ethnic minority adolescents in China during the pandemic

To investigate the potential mediating effects of PTSS on the relationship between intrusive rumination and academic burnout, we tested its role using Model 4 of Hayes SPSS Process 3.4 Macro [55]. Based on the preceding results, we first entered sex and learning stage as control variables in the model.

**Table 3:** The mediating effect of PTSS

Predictor	M: PTSS			Y: Academic burnout		
	*β*	*SE*	*t*	*β*	*SE*	*t*
C_1_: Sex	0.02	0.06	0.38	-0.06	0.06	-1.06
C_2_: Learning stage	0.16	0.07	2.47^*^	0.62	0.07	9.53^***^
X: Intrusive rumination	0.49	0.03	17.10^***^	0.13	0.03	4.06^***^
M: PTSS				0.38	0.03	11.75^***^
*R²*	0.24			0.27		
*F*	97.75^***^			87.25^***^		

^*^p<0.05, ^***^p<0.001

Table 3 displays the results of the mediation model test, which examined the mediating role of PTSS between intrusive rumination and academic burnout in adolescents, as well as the total effect of intrusive rumination on academic burnout. Intrusive rumination positively predicted PTSS (*β* = 0.49, *p*<0.001, 95%CI = 0.46, 0.55), and PTSS had a significant positive predictive effect on academic burnout (*β* = 0.38, *p*<0.001, 95%CI = 0.31, 0.44). The indirect effect size of PTSS is 0.19 (95%CI = 0.14, 0.24), accounting for 58.51% of the total effect.

Next, we analysed the mediating effect of the PTSS and the moderating effect of cognitive reappraisal using Model 59. As shown in Fig. 1, Model 59 consists of the predictor variable X (intrusive rumination), mediating variable M (PTSS), moderating variable W (cognitive reappraisal), and outcome variable Y (academic burnout). Similarly, sex and learning stage were the control variables.

**Table 4:** The mechanism of Intrusive rumination on academic burnout

Predictor	M: PTSS			Y: Academic burnout		
	*β*	*SE*	*t*	*β*	*SE*	*t*
C_1_: Sex	0.04	0.06	0.67	-0.03	0.06	-0.56
C_2_: Learning stage	0.15	0.07	2.31^*^	0.61	0.06	9.52^***^
X: Intrusive rumination	0.50	0.03	17.33^***^	0.16	0.03	4.89^***^
M: PTSS				0.36	0.03	11.09^***^
W: Cognitive reappraisal	-0.11	0.03	-3.91^***^	-0.20	0.03	-7.26^***^
X×W	-0.01	0.02	-0.36	-0.05	0.03	-1.98^*^
M×W				0.04	0.03	1.32
*R²*	0.25			0.31		
*F*	62.55^***^			60.78^***^		

^*^p<0.05, ^***^p<0.001

**Fig. 2:**
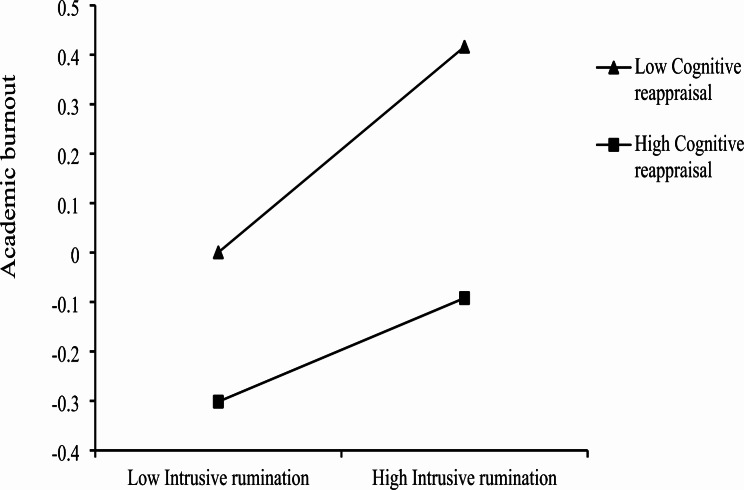
Moderating effect of cognitive reappraisal in the relationship between intrusive rumination and academic burnout

Table 4 displays cognitive reappraisal played a moderating role in the direct impact pathways of intrusive rumination and academic burnout. The interaction coefficient between intrusive rumination and cognitive reappraisal was − 0.05 (*p*<0.05), indicating that cognitive reappraisal attenuated the relationship between intrusive rumination and academic burnout. To provide a clearer understanding of the moderating effect of cognitive reappraisal, a simple slope graph was created to show how the effects of intrusive rumination on academic burnout were regulated by different levels of cognitive reappraisal. The mean of cognitive reappraisal plus (minus) one standard deviation was defined as a high (low) cognitive reappraisal. Figure 2 illustrates that intrusive rumination was significantly associated with academic burnout among adolescents, while cognitive reappraisal was low (*β* = 0.21, *p*<0.001) or high (*β* = 0.10, *p*<0.001). As the level of cognitive reappraisal increased, the predictive effect of intrusive rumination on academic burnout tended to decrease.

## Discussion

### Intrusive rumination and academic burnout: the role of PTSS and cognitive reappraisal

This study aimed to reveal the psychological characteristics of adolescents in Wenchuan, China during the COVID-19 pandemic, focusing on intrusive rumination, academic burnout, PTSS, and cognitive reappraisal.

The results indicated that the detection rate of PTSS among the participants was lower than that reported among adolescents in other parts of China during the COVID-19 pandemic [56] and the prevalence of PTSD in the general population mentioned by Breslau [57]. This finding may be attributable to the cultural particularities of the region, such as ‘Tibetan Buddhism’ [41] and ‘Qiang folk beliefs’ [43], which may have fostered a positive and optimistic cognitive mindset among young people. Although their learning status was affected to some extent by the public health emergencies, their mental health was generally better than expected. Furthermore, the study investigated sex, learning-stage, and ethnic differences in four psychological variables. Intrusive rumination differed significantly only by sex and learning stage. Males exhibited higher levels of intrusive rumination than females, which could be attributed to the tendency of males to suppress and mask negative emotional experiences [32]. Intrusive rumination levels were higher in junior high school students than in high school students, perhaps because students become more coping and regulating with age. Academic burnout was found to differ significantly only in the learning phase, with high school students exhibiting higher levels of burnout than junior high school students. This finding is consistent with previous research on the learning status of adolescents during the pandemic [16]. This may be because high school students experience more study pressure and significant academic burnout as the learning stage progresses. No significant differences were observed in the use of PTSS and cognitive reappraisal strategies regarding sex, learning stage, or ethnicity. Moreover, this study found no significant differences in intrusive rumination, academic burnout, PTSS, or cognitive reappraisal scores among Han, Tibetan, and Qiang adolescents in the Wenchuan area. This result may be attributed to the region’s multiethnic social and cultural background, which has fostered continuous contact and interaction among different ethnic groups, promoting social and cultural integration.

Based on previous research, this study investigated the mechanism of adolescent academic burnout in Wenchuan, China, during the COVID-19 pandemic. Consistent with Hypothesis 1, the findings confirm that during the COVID-19 pandemic, intrusive rumination significantly predicted academic burnout among ethnic minority adolescents in China. This result aligns with that of previous research conducted during the COVID-19 pandemic, indicating that higher levels of rumination are associated with a higher risk of burnout [58]. It may be because trauma-related rumination, to some extent, increases academic pressure and negative emotional perception among adolescents [19, 20], which, in turn, diminishes their academic interest and self-confidence, thereby promoting the onset and exacerbation of academic burnout. Additionally, as mentioned in the resource allocation theory, this may be related to the easy occupation and depletion of individual cognitive resources by stressful events [18]. Individuals may reduce their engagement in learning, leading to academic burnout characterised by avoidance.

Furthermore, the research results reaffirm the impact of trauma-induced intrusive rumination on various aspects of adolescent mental health [17, 59]. In line with Hypothesis 2, the results revealed that PTSS was crucial in mediating the relationship between intrusive rumination and academic burnout. This study demonstrated that intrusive rumination not only exerts a significant positive predictive effect on academic burnout but also indirectly exacerbates academic burnout through PTSS. This aligns with prior research indicating a positive correlation between intrusive rumination and PTSS [60, 61], whereby individuals experiencing trauma tend to engage in intrusive rumination and focus on negative aspects, leading to the development and persistence of post-traumatic stress symptoms. In addition, the study results are consistent with previous empirical research on ethnic minority adolescents in China and adolescents during the COVID-19 pandemic [39, 62], demonstrating a positive predictive relationship between PTSS and academic burnout. Regarding Hypothesis 3, cognitive reappraisal can significantly and negatively predict levels of academic burnout in adolescents. This supports the theory of self-regulation, which involves individuals managing, adjusting, and controlling their emotions, thoughts, and behaviours to cope with various situations and challenges. Cognitive reappraisal, as a crucial strategy in self-regulation, helps improve individuals’ experiences of academic stress and reduces negative emotional perceptions [63, 64], thereby alleviating academic burnout. These results indicate that cognitive reappraisal only plays a regulatory role in the direct impact of intrusive rumination on academic burnout. Specifically, as the level of cognitive reappraisal increased, the effect of intrusive rumination on academic burnout decreased, partially supporting Hypothesis 3. This finding aligns with Gross’s [65] proposed function of cognitive reappraisal, which posits that emotional responses are reduced by altering the perception of emotional events at the level of personal meaning. The generation and development of PTSS may involve factors other than individual cognition and emotion; the moderating role of cognitive reappraisal in the other two indirect pathways was not obvious.

Based on the findings of this study, we make three main recommendations. First, researchers should explore the reasons behind the lower incidence of PTSS in adolescents in this ethnic region during the crisis and utilise the advantages and roles of these factors. For example, the cultural values of ‘optimism and open-mindedness’ and ‘peaceful view of life and death issues’ in Tibetan culture, and ‘fearless of difficulties’ and ‘awe and conformity to nature’ in Qiang culture, alongside unique minority resources like ethnic dances and religious activities, are worth fully utilising. These resources can contribute to developing positive concepts and self-regulation abilities among young people, thus promoting the construction of public psychological service systems in ethnic areas. Second, considering the substantial mediating roles observed, future research and practice must concentrate on the development and implementation of targeted assessments and intervention programs aimed at identifying and addressing PTSS among adolescents. These programs should encompass effective screening methods to detect PTSS early and tailored interventions designed to alleviate and prevent PTSS and mitigate the risk of academic burnout in adolescents. Third, based on the moderating role of cognitive reappraisal, families and schools should form alliances to strengthen the emotion regulation education and guidance of students, especially in the context of the crisis. They should focus on the cognitive reconstruction of adolescents to prevent and alleviate psychological problems, such as academic burnout caused by adverse cognitive processes such as intrusive rumination.

### Limitations and future directions

This study had some limitations. First, the cross-sectional design used in this study makes it difficult to accurately investigate the causal relationship between variables and make long-term judgments about the changing trends in the relationship between variables. Future research should explore this further through longitudinal studies. Second, this study explored only one emotion regulation strategy, cognitive reappraisal, and other emotion regulation strategies and methods are worth analysing and testing to provide better guidance for practice. Third, the research sample only included adolescents from Tibetan and Qiang settlements in Southwest China. Therefore, it remains unclear whether these results generalise to other ethnic minorities from different cultural backgrounds in China. Further verification is required to test the generalisability of our findings.

### Research significances

Notwithstanding the aforementioned limitations, this study theoretically revealed the psychological characteristics and interaction mechanisms of adolescents in ethnic minority areas of China in terms of cognition, emotion regulation, PTSS, and academic performance during the COVID-19 pandemic. This study adds to the literature on ethnicity and the COVID-19 pandemic. Moreover, it establishes a foundation for subsequent practical interventions in this domain.

## Conclusions

In this study, we aimed to reveal the psychological characteristics of adolescents in ethnic minority areas of China during the COVID-19 pandemic and elucidate the underlying mechanisms of academic burnout. This study aimed to provide a theoretical and practical basis foundation for understanding adolescent psychological and behavioural problems in the context of trauma. This study yields the following key findings:

(1) In general, adolescents in ethnic minority areas of China exhibited a lower rate of PTSD during the pandemic. (2) Intrusive rumination during the pandemic directly predicted academic burnout in adolescents and indirectly exacerbated academic burnout by causing or worsening PTSS. (3) Cognitive reappraisal significantly and negatively predicted academic burnout among adolescents. This weakens the relationship between intrusive rumination and academic burnout among adolescents. With higher levels of cognitive reappraisal, intrusive rumination had less effect on academic burnout. These findings have significant theoretical and practical implications for addressing adolescent psychological and behavioural problems in the context of trauma.

## Data Availability

The datasets used and analyzed during the study are available from the corresponding author on reasonable request.

## List of abbreviations

COVID 19: Coronavirus Disease 2019
PTSD: Post traumatic stress disorder
PTSS: Post traumatic stress symptoms
